# Advances in long-read single-cell transcriptomics

**DOI:** 10.1007/s00439-024-02678-x

**Published:** 2024-05-24

**Authors:** Pallawi Kumari, Manmeet Kaur, Kiran Dindhoria, Bruce Ashford, Shanika L. Amarasinghe, Amarinder Singh Thind

**Affiliations:** 1 Institute of Microbial Technology, Council of Scientific and Industrial Research, Chandigarh, India; 2 Illawarra Shoalhaven Local Health District (ISLHD), NSW Health, Wollongong, NSW Australia; 3 Monash Biomedical Discovery Institute, Monash University, Clayton, VIC 3800 Australia; 4 Walter and Eliza Hall Institute of Medical Research, 1G, Royal Parade, Parkville, VIC 3025 Australia; 5 The School of Chemistry and Molecular Bioscience (SCMB), University of Wollongong, Loftus St, Wollongong, NSW 2500 Australia

## Abstract

Long-read single-cell transcriptomics (scRNA-Seq) is revolutionizing the way we profile heterogeneity in disease. Traditional short-read scRNA-Seq methods are limited in their ability to provide complete transcript coverage, resolve isoforms, and identify novel transcripts. The scRNA-Seq protocols developed for long-read sequencing platforms overcome these limitations by enabling the characterization of full-length transcripts. Long-read scRNA-Seq techniques initially suffered from comparatively poor accuracy compared to short read scRNA-Seq. However, with improvements in accuracy, accessibility, and cost efficiency, long-reads are gaining popularity in the field of scRNA-Seq. This review details the advances in long-read scRNA-Seq, with an emphasis on library preparation protocols and downstream bioinformatics analysis tools.

## Introduction

During the past two decades, short-read bulk RNA-Seq (NGS) has been widely used for biological research, particularly because of its cost-effectiveness in quantifying the expression of genes on a genome-scale and identifying novel genes, in comparison to microarrays (Conesa et al. 2016; Rao et al. 2019; Thind, et al. 2021). However, due to the innate short 150-300 bp fragment length of reads, short reads still struggle to identify complex transcriptome events such as alternative splicing and gene fusions that result in incomplete transcript reconstruction thus making it challenging to accurately analyse data on the transcriptome level (Midha et al. 2019; Deshpande et al. 2023) (Steijger et al. 2013; Angerer et al. 2017; Lähnemann et al. 2020; Adil et al. 2021). In comparison, recent advancements in long-read technologies such as the ones that use voltage-driven protein nanosensor technology (e.g., Oxford Nanopore Technology; ONT) or fluorescent sequential binding (e.g., Pacific BioSciences; PacBio) (Mantere et al. 2019; MacKenzie and Argyropoulos 2023) allow construction of complete transcript isoforms due to the possibility of reads spanning over entire transcripts (Payne et al. 2019; Wang et al. 2021a). A host of studies using long-read sequencing have shown that there is more isoform diversity in the human genome than anticipated (Zhou et al. 2019; Wright et al. 2022; Heberle, et al. 2023; Page, et al. 2024). Such studies show the role of these novel isoform in various diseases (Ray et al. 2020; Huang et al. 2021; Veiga, et al. 2022; Paoli-Iseppi, et al. 2024). Veiga, et al. (2022) employing long-read sequencing, not only pinpointed approximately 300 breast tumor-specific splicing events, showcasing the technology's enhanced splicing event detection, but also highlighted that a few of these events are significantly correlated with patient survival. Apart from generating longer reads than traditional NGS, long-read technologies also possess the ability to give technology-specific novel insights, such as RNA modifications and detection of expressed mutations in RNA for genotyping.

Bulk RNA-Seq, whether short or long-read fails to capture cellular heterogeneity and RNA extracted from a mixed cell population only provides an average gene expression profile (Thind, et al. 2021). This averaging can mask or dilute the expression signals of specific cell types, leading to challenges in accurately identifying differential gene expression between different conditions or groups. This limitation is particularly evident in cancer studies, where tumor samples often contain a mixture of tumor cells and surrounding healthy cell types. Only samples with high tumor purity can yield accurate detection of gene expression signals specific to the tumor cells, while lower purity samples may obscure these signals within the averaged expression profile. The discovery of single-cell transcriptomics (scRNA-Seq) in 2009, offered greater insight into complex biological system including tumour heterogeneity (Hwang et al. 2018), novel cell types (Stuart, et al. 2019), drug-resistant clones (Adewale 2020), role of sub-cellular population in disease progression (Shi et al. 2023), treatment response (Volden and Vollmers 2022), and overall cellular function (Hazzard et al. 2022; Thijssen et al. 2022). Over time, new single-cell library preparation protocols and analysis methods have been developed with the aim of reducing sequencing costs and/or increasing throughput (Healey et al. 2022; Yang, et al. 2023). Short read single cell sequencing is being done more frequently in the past decade than long read sequencing, primarily due to lower cost and faster turnaround times.

Similar to short-read bulk RNA-Seq, short-read single-cell RNA-Seq has challenges in identifying isoforms and gene fusions at the single-cell level (Steijger et al. 2013; Angerer et al. 2017; Lähnemann et al. 2020; Adil et al. 2021). Other issue includes incomplete 3′-UTR annotations due to its bias towards the 3′ end (Healey et al. 2022), which could give misleading impressions about their expression in different cell types. Cell type identification analysis relies on finding patterns in highly variable genes to cluster cell types (Stuart et al. 2019). If these crucial genes are systematically missing, it can lead to mistakes in analysis and obscure the true biological picture. In contrast, long-reads have allowed capturing the isoform diversity even at single-cell resolution which can improve 3′-UTR annotations, hence cell type identification (Adewale 2020; Volden and Vollmers 2022; Shi et al. 2023; Healey et al. 2022). Despite the initial limitations such as higher cost, and elevated error rates of early long-read technologies, their popularity is now growing for single-cell applications with advancements in the field. For example, Thijssen et al. (2022) applied a novel nanopore single-cell approach to identify mutations and alternative transcripts in specific sub-clones of the tumour at relapse. In another study, Hazzard et al. (2022) used PacBio sequencing with 10X Chromium scRNA-Seq libraries to reveal the isoform diversity of *Plasmodium vivax* transcripts at different developmental stages. Healey et al. (2022) conducted single-cell isoform sequencing alongside single-cell RNA sequencing, quickly enhancing 3′-UTR annotations. They demonstrated that gene models derived from a minimal embryonic single-cell isoform sequencing dataset retained 26.1% more single-cell RNA sequencing reads compared to gene models from Ensembl alone, consequently amplifying the reads identified for numerous genes. Further, long-read scRNA-Seq can accurately predict gene fusion events at single cell level (Yang, et al. 2023) and facilitate the identification structural variants (Mahmoud et al. 2019). Subsequently, PacBio has introduced MAS-Iso-seq, a new programmable concatenation of DNA fragments into long library molecules which enables single-cell sequencing with high throughout (14–18 fold increase) compared to their Iso-Seq approach (Al’Khafaji et al. 2023). However, these technologies are yet to overcome the limitations of traditional NGS scRNA-Seq across several domains including higher throughput (Boldogkői et al. 2019; Amarasinghe et al. 2020; Oikonomopoulos et al. 2020).

**Fig. 1:**
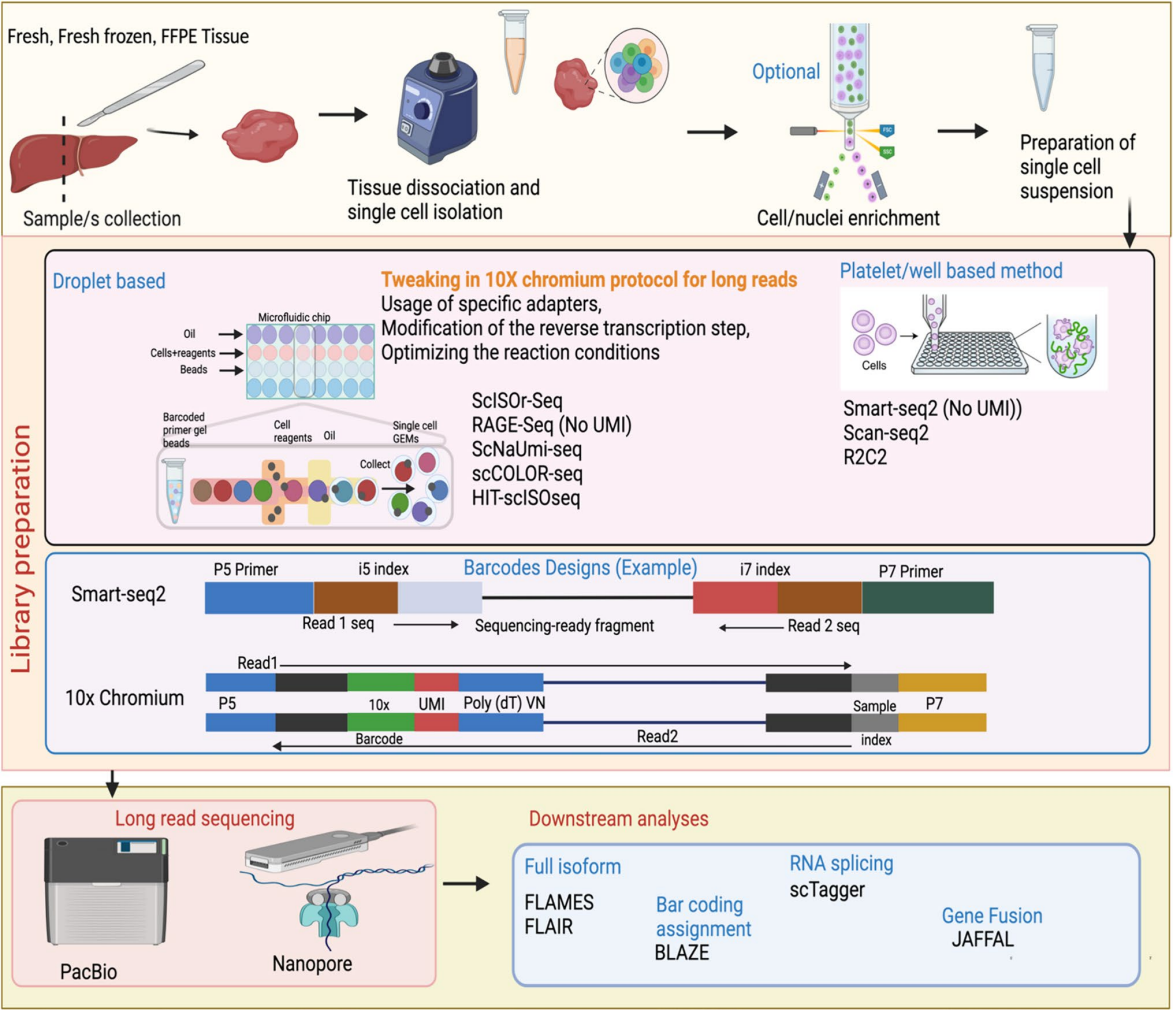
Process of long-read scRNA-Seq data generation and analysis. The tissue is collected, homogenized, and enriched for the preparation of the selected single-cell suspension. The library is prepared by droplet-based and platelet-based approaches with the help of specifically designed adaptors. The long-read scRNA-Seq sequencing is generally carried out by either PacBio or Nanopore sequencing platforms. The downstream processing of the sequencing output files is then analysed downstream by available bioinformatics tools

In this review, we aim to give a snapshot of available long-read sequencing technologies, current long-read single-cell isolation protocols, and downstream bioinformatics analysis methods long-read scRNA-Seq data (Fig. 1).

## Currently available long-read sequencing technologies

PacBio and ONT, being the two dominant platforms in long-read technologies, offer a range of platforms for sequencing, e.g., REVIO, Sequel, Sequel II, SEQUEL from PacBio and MinION, GridION, and PromethION devices from ONT (Amarasinghe et al. 2020). PacBio relies on its proprietary single-molecule, real-time (SMRT) sequencing technology that uses a sequencing-by-synthesis method. PacBio sequencing technology uses HiFi reads that provide accuracy of 99.9%, on par with short reads and Sanger sequencing (Logsdon et al. 2020). With redesigned SMRT cell and improved sequencing chemistry than its predecessors, Sequel II now allows generation of data up to 10 Gb per SMRT cell (Logsdon et al. 2020). Additionally, Sequel II circular consensus sequencing (CCS) improves the read accuracy by sequencing the same SMRT molecule iteratively (Dijk 2023). The Sequel II with CCS has the highest accuracy of the three PacBio instruments, with accuracy rates approaching 99.9%. It is recorded that with the novel MAS-Iso-Seq approach, Sequel-II can produce up to 40 million cDNA reads per run (Al’Khafaji et al. 2023).

ONT uses a nanopore-based sequencing method (Wang et al. 2021a). This concept revolves around a process where each DNA or RNA molecule travels through a nanometre scaled protein pore in an electrophysiological solution. As it passes, ions move with it, their concentrations varying according to the molecule's nucleotide composition (Midha et al. 2019). This activity results in identifiable patterns of current fluctuations that correspond to the sequence of nucleotides. By analysing the digitalised data of these current levels, pretrained artificial neural networks can accurately predict the resulting sequence. Since this approach does not rely on imaging required by popular sequencing-by-synthesis methods, sequencing devices could be significantly reduced in size. Having the ability to be either portable with low throughput and low cost or high throughput due to large number of flow cells makes ONT technology popular in metagenomic and microbial research (Zhu 2020) as well as human genome-based research (Bowden et al. 2019). Currently, there are attempts to figure out the usefulness of shallow sequencing (Milanez-Almeida et al. 2020; Mock et al. 2023) or targeted sequencing (Gilpatrick et al. 2020; McClinton et al. 2023) that would allow low throughput ONT machines (e.g., minION) to be used in clinical setting.

ONT PromethION generates more reads per flow cell than the PacBio Sequel II and offers the longest reads, with some reads exceeding 2 Mb in length (Payne et al. 2019). ONT has a higher error rate compared to PacBio, with an error rate of 10–15% (Table 1), although this can be mitigated by using base calling algorithms and consensus approaches (Fu et al. 2019). The cost per base for ONT is lower than PacBio, although the difference in cost can vary depending on the specific application and sequencing strategy (Dijk 2023).

**Table 1:** Features of available long-read sequencing instruments

	Sequel I (PacBio)	Sequel II (PacBio)	PromethION (ONT)	MinION (ONT)
Methods used for sequencing	Sequencing by synthesis	Sequencing by synthesis	Nanopore based Sequencing	Nanopore based Sequencing
Average range of read length	10 k–100 k bases	15 k–100 k bases	5 k–1 M bases	1 k–10 k bases
Accuracy	> 99.9%	> 99.9%	97–99%	95–99%
Throughput	Lower than both Sequel II and ONT	Higher throughput than Sequel I	High Throughput	–

### Long read sequencing for RNA modifications and expressed mutations in RNA for genotyping

Long-read technologies not only produce longer reads compared to traditional NGS methods but also offer unique, technology-specific insights. For example, ONT can potentially detect the RNA modifications, such as m6A and pseudo uridine, directly within RNA molecules (Leger et al. 2021) (Felton, et al. 2022; Dorney, et al. 2023). Traditional methods like mass spectrometry, NGS and biophysical assays that are widely used to identify RNA modifications have their own drawbacks (Helm and Motorin 2017). While mass spectrometry provides limited site-specific information, NGS can only detect a limited number of abundant modifications, and biophysical assays share both these limitations (Helm and Motorin 2017). Nanopore direct RNA sequencing has the ability to directly sequence entire, unaltered RNA molecules without requiring reverse transcription (RT) or amplification steps (Garalde et al. 2018). This innovation has revealed a hidden advantage: Nanopore data inherently captures information about modifications present within the RNA molecules themselves (Smith et al. 2019; Workman et al. 2019). This advantage undoubtedly unlocks a new level of detail in the field of epi-transcriptomics. Many software tools are currently being developed for the accurate detection of these RNA modification from ONT data (Furlan et al. 2021; Stephenson, et al. 2022).

PacBio has the advantage of generating more accurate long-reads due to its circular consensus sequencing. In brief, the PacBio libraries prepared in their proprietary SMRT Bell format will get repeatedly read by the sequencer. This enables the sequencing of the same read repeatedly which will then get collapsed into a random “error-free” consensus sequence during downstream analysis. This approach, however, has the drawbacks of having to use more storage for storing raw reads and making sequencing more expensive and time consuming than ONT. Nevertheless, PacBio RNA sequencing has accurately allowed the detection of expressed mutations in RNA for genotyping and neo-antigen discovery (Cavelier et al. 2015).

## Tissue dissociation and library preparation for long-read scRNA-Seq

### Suitability of various tissues for scRNA-Seq libraries preparation

Generating a homogeneous single-cell suspension from the tissue of interest is the first step of any scRNA-Seq pipeline (Gross et al. 2015). To do this isolation of viable cells from the target tissue at an optimal dissociation level (i.e., a balance between releasing cell types that are difficult to dissociate while avoiding damage to those that are fragile) is required (Denisenko et al. 2020; Burja et al. 2022). The choice of input tissue type suitable for scRNA-Seq depends on several factors, including the availability of adequate amounts of high-quality tissue, the goal of the experiment, and the method used for tissue preservation. Fresh-frozen tissue is often considered the gold standard for scRNA-Seq experiments as it provides the highest quality RNA (Yin et al. 2021). Harvested tissue is immediately frozen in liquid nitrogen after collection and stored at −80 °C to minimize RNA degradation. Whole-cell scRNA-Seq approaches and single-nucleus RNA sequencing protocols (snRNA-Seq) are two widely used approaches of sc transcriptomics for various tissue types (Slyper et al. 2020). Whole-cell scRNA-Seq approaches offer several advantages over snRNA-Seq, including comprehensive transcriptome analysis, the ability to study cytoplasmic transcripts, cell-type-specific gene expression patterns, intracellular interactions, and higher-quality RNA (Jovic et al. 2022). However, it has several disadvantages compared to snRNA-Seq, including limited resolution of nuclear gene expression, higher levels of background noise (due to transcripts from the cytoplasm and organelles), RNA degradation & more difficult tissue preparation (isolation of whole cells is more difficult than nuclei and may cause more mechanical damage), and challenges studying specific cell types or subpopulations of cells (Nguyen et al. 2018) (Bakken et al. 2018). According to a study conducted on the human liver, snRNA-Seq was more effective in detecting many other cell subtypes, including cholangiocytes and mesenchymal cells. These cell types are typically challenging to distinguish by scRNA-Seq alone (Andrews et al. 2022). Nuclei isolation can be used to analyse gene expression from fixed and/or formalin-fixed paraffin-embedded (FFPE) tissue, which may not be possible with whole-cell scRNA-Seq approaches (Rousselle et al. 2022). Nuclei isolation is also a useful approach for studying rare cell types (Wu et al. 2019). Therefore, it is important to consider the specific goals of the experiment and the quality of the tissue available when deciding which approach to use (Sant et al. 2023).

snRNA-Seq protocols can be applied to snap-frozen samples, avoiding many of the dissociation-related artifacts (Krishnaswami et al. 2016; Lake et al. 2016). It is more difficult to isolate live single cells upon thawing complete tissue that has been frozen, hence it is preferable in this situation to remove the nuclei from the tissue before it is snap frozen. Single-nuclei methods also permit the profiling of nuclei from large cells (> 40 μm) that do not fit through the microfluidics. When there is availability for fresh tissue, manual isolation is a suitable platform for undertaking scRNA-Seq. SnRNA-Seq utilizing a droplet-based technique is appropriate when there are frozen samples available, and isolation of viable whole cells is difficult (Martelotto 2019). Compared to isolating cells from fresh tissues, dissected tissues are frozen using either the snap-freeze or slow-freeze techniques that reduce overall cell recovery. If a well-optimized tissue dissociation protocol is already available, then dissociation followed by cryopreservation is preferred. However, as per 10 × Genomics recommendation, if the tissue dissociation protocol is not optimized, then it may be better to snap-freeze the tissue whole (10xGenomics, Are fresh frozen tissue samples compatible with Single Cell RNA sequencing? https://kb.10xgenomics.com/hc/en-us/articles/360019890851-Are-fresh-frozen-tissue-samples-compatible-with-Single-Cell-RNA-sequencing-, Accessed Sept 2023).

Formalin-fixation (and paraffin-embedding–FFPE) of tissue is a commonly used preservation method, but it is not ideal for scRNA-Seq experiments due to the potential for RNA degradation and crosslinking during the fixation process (Chung, et al. 2022). However, the greatest collection of clinically documented human samples can be found as FFPE tissue. FFPE-fixed single cell sequencing can be attempted using labour-intensive, slow, insensitive, and low-resolution techniques, making it difficult to fully utilize the huge research and clinical potential of these samples. Despite these challenges, some scRNA-Seq experiments have been successfully performed on FFPE tissue (Vallejo, et al. 2022). FFPE tissue types suitable for scRNA-Seq include cancer tissues, where the goal is to study the heterogeneity of tumour cells, and tissues with low cell numbers or rare cell types, where preservation in FFPE may be the only option (Gao et al. 2020). Single nuclei extraction from FFPE specimens can be profiled using the effective and sensitive high-throughput technique known as single nuclei pathology sequencing (snPATHO-Seq) (Vallejo, et al. 2022). The 10 × Genomics probe-based technology that targets the entire transcriptome is combined with an improved protocol for extracting nuclei from archived samples to undergo snPATHO-Seq. In summary, fresh-frozen tissue is considered the best choice for scRNA-Seq experiments, but FFPE tissue can also be used.

### Library preparation for long-read scRNA-Seq

Like other scRNA-Seq, even for long-read sequencing technologies like ONT or PacBio, both unique molecular identifiers (UMI) and barcodes are typically used in combination (but not always) to enable accurate and reliable quantification of gene expression at the single-cell level (Table 2). UMIs allow for the reduction of PCR and sequencing errors, while barcodes enable the identification of individual cells and their respective RNA molecules. Together, these features facilitate the generation of accurate and high-quality scRNA-Seq data. However, the assignment of barcodes and UMIs is more challenging for long-reads than for short-reads because long-read sequencing platforms have a higher error rate. Long-read can span multiple genes or transcripts, which can lead to ambiguity in assigning UMIs to specific genes or transcripts. Dropout occurs when a transcript is not detected in a particular cell due to technical noise or low sequencing depth. This can occur more frequently with long-read sequencing technologies due to the increased technical noise associated with these methods because they generate reads that are more error-prone due to factors such as polymerase errors, signal variability, and template degradation (Amarasinghe et al. 2020; Prawer, et al. 2023). The advanced protocols have addressed library preparation challenges in single-cell long-read sequencing by introducing innovative strategies for error correction and increasing base accuracy (Table 2). This advancement overcomes the issues associated with barcode and UMI assignment in long-read sequencing platforms, ultimately providing more accurate and robust results for single-cell analysis than before (Figure 3).

**Table 2:** Currently available long-read scRNA-Seq library preparation protocols

Protocol	Library preparation (Base)	Sequencing	Limitations	Advantage	Features	References
Smart-seq2	Smart-seq2	Long-read	Lower cellular throughput, higher cost	Suitable for analysing rare cell types with low input amounts of RNA; allows for the detection of low-abundance transcripts. No UMIs incorporated	Good sensitivity with full-length coverage.; no UMI	Picelli et al. (2013)
R2C2	Tn5Prime method (modification of the Smart-seq2)	Long-read (ONT)	Require sufficient sequencing coverage to call consensus reads	Increase base accuracy of cDNA sequences (ONT) by circular consensus principle	Achieved 96% sequencing accuracy, this still only translated to 72% of barcodes demultiplexing correctly,	Volden et al. (2018)
ScISOr-Seq	Adopt 10 × Genomics	Long-read + short-read (Illumina)	No correction for UMI sequencing errors; Low sequencing depth or high cost	Use Illumina sequencing data of the same libraries to guide cell barcode assignment	PacBio’s Iso-seq has been further adapted to use the 10 × Genomics platform	Gupta et al. (2018)
RAGE-Seq	Adopt 10 × Genomics;	Long-read + short-read (Illumina)	Focuses on specific transcripts rather than the whole transcriptome; low recovery of cell barcodes due to the higher error-rate of base-called nanopore sequencing data	Cheaper compared to smart-seq2. Reports antigen receptors, ScISOr-Seq don’t report it	(1) No UMIs, (2) use Illumina sequencing data of the same libraries to guide cell barcode assignment	Singh et al. (2019)
ScNaUmi-seq	Adopt 10 × Genomics	Long-read + short-read (Illumina)	Require Illumina reds for error correction; low capture efficiency; requires saturated sequencing to error correct UMI sequences, which increases the sequencing cost	Unlike R2C2 and ScISOr-Seq, this method Correct UMI sequencing errors	Use UMI;	Lebrigand et al. (2020)
SCAN-seq	Modified Smart-seq2	Long-read	The original method does not implement any specific strategy to overcome the high error rate of nanopore sequencing	Better cells capture efficiency (80%), Capture thousands of unannotated transcripts of diverse types	Need small amount of starting material	Fan et al. (2020)
FlsnRNA-Seq	10 × Genomics	Long-read (ONT)	snRNA-Seq relative to scRNA-Seq has relatively low RNA content in nuclei may leads to lower transcripts recovered per cell and reduces the resolving power of the resulting snRNA-Seq data	Capture isoform diversity at the single-nucleus level	Original study is plants based	Long et al. (2021)
scCOLOR-seq	Drop-seq	Long-read (ont)	Low Cell capture efficiency (10%)	Overcome the low base-calling accuracy of Oxford Nanopore sequencing; provides a simplified and more robust method to perform quantitative long-read transcript sequencing on large numbers of cells	Error correction of single cell sequencing barcodes, with over 80% recovery of reads when using an edit distance of 7, or over 60% recovery when using a conservative edit distance of 6. Uniquely, UMIs can be deduplicated with a high level of accuracy, approaching 100% in simulated data	Philpott et al. (2021)
LR-Split-seq	Microfludic	Long-read + short-read (Illumina)	–	No cell/droplet handling instrument required	Allow flexible study design	Rebboah et al. (2021)
R2C2 with the 10X Genomics platform	10X Genomics platform	Long-read	–	Higher throughput than R2C2: 12 million full-length cDNA molecules (3 k cells)	Independent from Illumina data	Volden and Vollmers (2022)
SCAN-seq2	Modified Smart-seq2	Long-read (ONT)	–	High-throughput, high-sensitivity Claimed	First-strand cDNAs of every 32 single cells with different 3′ barcodes were pooled together after reverse transcription. By introducing 24-nt barcodes to the PCR primers, different 5′ barcodes were added during PCR amplification	Liao et al. (2023)
HIT-scISOseq	PacBio’s Iso-seq has been further adapted to use the 10 × Genomics platform	Long-read	–	High read throughput, yields > 10 million high-accuracy long-reads in a single PacBio Sequel II SMRT Cell, high-accuracy > 99.99% accuracy and specificity provided by scISA-Tool		Shi et al. (2023)
MAS-ISO-seq		Long-read (PacBio)	Greater sequencing depth may be required to ensure sufficient power for downstream single-cell analyses	Deplete TSO priming artifacts boost the throughput (cDNA reads) > 15-fold increase over CCS-corrected read counts	A programmable concatenation of cDNAs	Al’Khafaji et al. (2023)

### Handling library preparation artifacts

**Fig. 2:**
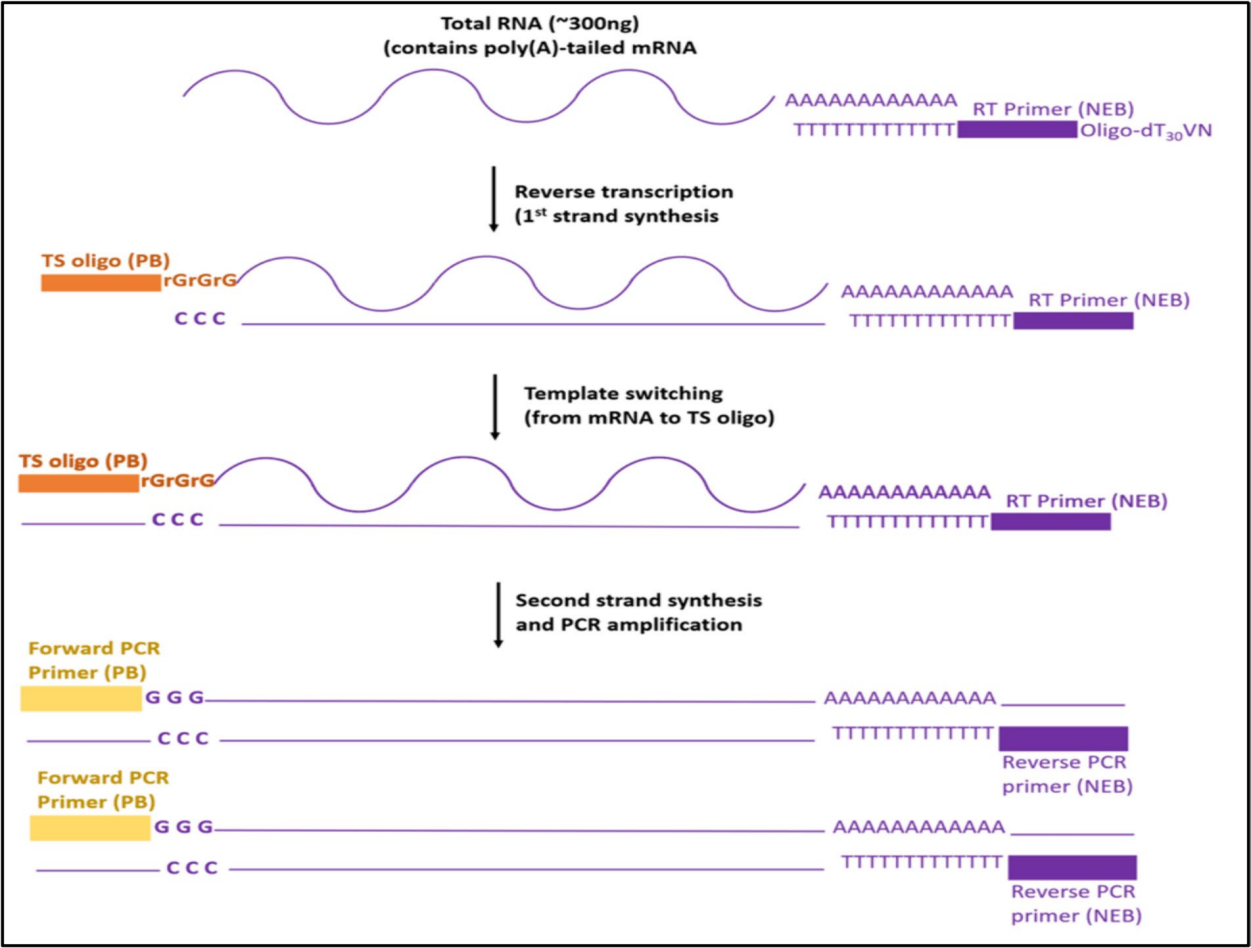
Summary of Template Switching Oligo (TSO) mechanism. The violet and orange rectangles correspond to amplification primer tags. During first-strand synthesis priming with an oligo dT primer is done to capture polyadenylated transcripts, upon reaching the 5′ end of the RNA template a few additional nucleotides (mostly deoxycytidine) are added to the 3′ end of the newly synthesized cDNA strand. The simple version of a TS Oligo is a DNA oligo sequence that carries 3 riboguanosines (rGrGrG) at its 3′ end. The complementarity between these consecutive rG bases and the 3′ dC extension of the cDNA molecule empowers the subsequent template switching

The template switching oligo (TSO) artifact is a potential issue in single-cell RNA sequencing (scRNA-Seq) methods that use the Smart-seq2 protocol or other similar protocols. TSO are short oligonucleotides that are added during the reverse transcription step of scRNA-Seq library preparation to prime the synthesis of the complementary DNA (cDNA) strand from the template RNA (Fig. 2). The TSO artifact occurs when the TSO anneals to the cDNA strand synthesized during reverse transcription and acts as a template for the synthesis of a new cDNA strand, leading to the incorporation of extra nucleotides into the cDNA sequence (Picelli 2017). For example, a threshold can be set on the length of the reads to remove those that are likely to contain the TSO sequence. Additionally, statistical models can be used to infer the correct cell barcodes based on the distribution of read counts and UMIs across the barcodes (You et al. 2023).

Unlike the Smart-seq2 protocol, the 10 × Genomics Chromium system uses a unique barcoding technology to capture the RNA molecules. In single-cell 3' RNA sequencing using 10 × Genomics, a small amount of the libraries is expected to have the TSO sequence at the beginning of the second read (10xGenomics, Why do a fraction of my Visium reads contain the Template Switch Oligo (TSO) at the beginning of Read 2? https://kb.10xgenomics.com/hc/en-us/articles/360041690731-Why-do-a-fraction-of-my-Visium-reads-contain-the-Template-Switch-Oligo-TSO-at-the-beginning-of-Read-2, Accessed Sept 2023). However, if a large fraction of the library has the TSO sequence, this could indicate a problem with the library preparation such as (a) Significantly shorter cDNA or cDNA degradation than expected before starting the reverse transcription reaction, (b) TSO sequence is not efficiently removed from the cDNA construct during library preparation (10xGenomics, Why do a fraction of my Visium reads contain the Template Switch Oligo (TSO) at the beginning of Read 2? https://kb.10xgenomics.com/hc/en-us/articles/360041690731-Why-do-a-fraction-of-my-Visium-reads-contain-the-Template-Switch-Oligo-TSO-at-the-beginning-of-Read-2, Accessed Sept 2023).

### Modified short-read scRNA-Seq library preparation protocols for long-reads

**Fig. 3:**
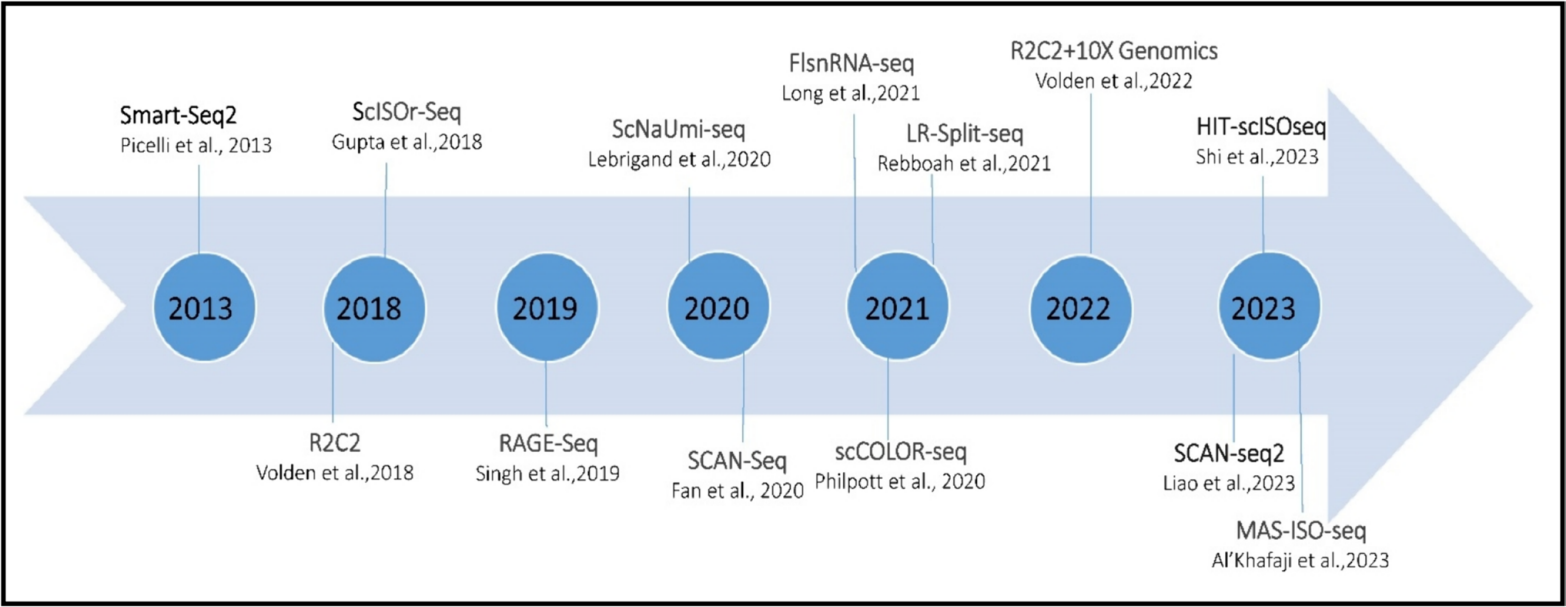
Evolution of long-read scRNA-Seq library preparation protocols

10 × Genomics, Smart-seq2, Tn5Prime (modified Smart-seq2) are some popular methods for scRNA-Seq library preparation. Tn5Prime is a modification of the Smart-seq2 protocol that uses the Tn5 transposase enzyme to simultaneously fragment and tag RNA molecules (Picelli et al. 2014; Picelli 2017; Cole et al. 2018). The main difference between 10 × Genomics scRNA-Seq and Smart-seq2 is in the way cells are processed and barcoded (Baran-Gale et al. 2018). In 10 × Genomics scRNA-Seq, cells are partitioned into droplets containing unique barcodes and molecular identifiers, enabling the capture and amplification of individual cells in a high-throughput manner. In contrast, Smart-seq2 uses a plate-based method in which individual cells are isolated and lysed in individual wells of a 96- or 384-well plates, and cDNA is synthesized using oligo (dT) primers. Furthermore, the original 10 × chromium platform does not generate long-reads but short-reads of typically 50–100 bp length from the 3' end of transcripts, while Smart-seq2 generates long-read (up to several kilobases) from the entire length of transcripts with higher sensitivity than 10x (Lebrigand et al. 2020; Tian et al. 2021). 10 × however has low cost per cell compared to Smart-seq2 (Fig. 3).

To be useful for long-read sequencing modifications have been made to the 10 × Genomics protocol with the use of specific adapters, modifications to the reverse transcription step, and optimisation to the reaction conditions etc. (Jabbari and Tian 2019; Lebrigand et al. 2020; Tian et al. 2021). For example, before PromethION flow cell (Oxford Nanopore) sequencing, Lebrigand et. al re-amplified the 10 × Genomics PCR product for eight cycles with different primers which contain Ns at the 5′ ends to avoid the preferential generation of reverse Nanopore reads (Lebrigand et al. 2020). Similarly, Jabbari et. al uses subsampling of 10 × Chromium generated single cell Gel Bead-in-Emulsions (GEMs) after reverse transcription for cDNA amplification and their protocol enables transcriptome sequencing of full-length cDNA from a flexible number of single cells captured on Chromium using long-read sequencers (Tian et al. 2021) and in some of their studies they followed standard 10 × Genomics user guide, with RT time increased to 2 h to potentially increase the reverse transcription of longer transcripts (Tian et al. 2021). The RAGE-Seq method, developed by Singh et al. (2019) builds upon the 10 × Chromium Single Cell 3' protocol with the addition of two extra PCR cycles. These cycles enable a 1:1 full-length cDNA split, with one portion utilized for Nanopore long-read sequencing and the other for short-read sequencing. Single-Cell Isoform RNA sequencing -Seq (ScISOr-Seq) (Gupta et al. 2018) is another example of long-read scRNA-Seq methods that have been developed by tweaking the 10 × Genomics protocol. ScISOr-Seq combines the 10 × Genomics protocol with Iso-Seq, a method developed by PacBio for full-length transcript sequencing. In ScISOr-Seq, single cells are captured using the 10 × Genomics protocol and then subjected to Iso-Seq library preparation to generate full-length cDNA sequences.

In terms of the modifications to the plate-based approaches, instead of the TSO used for the short-read protocol, a new template-switching oligonucleotide (LMO-PCR TSO) is used to capture, and reverse transcribe the mRNA in Smart-seq2. This TSO has a UMI, a poly (A) tail, and a hairpin adapter that facilitates the ligation of the cDNA to the sequencing adapter. The resulting cDNA library is amplified by long-range PCR to generate long fragments, which then can be sequenced on the long-read platforms. Some methods use a targeted transcript capture approach to enrich full-length transcripts such as HIT-scISOseq that uses biotinylated PCR primers, and a library preparation procedure that combines head-to-tail concatemeric full-length cDNAs into a long SMRTbell insert for high-accuracy PacBio sequencing (Shi et al. 2023). This allows for more efficient use of sequencing reads and reduces the sequencing cost, compared to other techniques that rely on the random sampling of transcripts.

### Handling sequencing depth

Single-cell sequencing typically requires a much greater sequencing depth compared to bulk long-read sequencing due to factors such as the desired number of reads per cell per sample, the isoforms diversity amongst cells (Sims et al. 2014; Rizzetto et al. 2017). It is known that increased read length improves read-to-transcript assignment (Chen, et al. 2021), which means that long-read sequencing may warrant less sequencing depth compared to short-read sequencing to gain the same isoform identification ability. For example, reads per cell obtained by Volden and Vollmers (2022) using the 10X Genomics platform and R2C2 were five times less than the number of short reads per cell recommended by 10X Genomics. Yet, this count of around 4000 R2C2 reads per cell managed to capture around 67% of the molecules present in a comprehensively sequenced Illumina dataset derived from the same cDNA. This level of coverage has been reported as adequate for cell type clustering and generating single-cell transcriptomes. However, based on the aim of study for sequence depth, one should consider factors such as sub-population rarity, desired resolution, and transcript complexity to measure etc. (Zhang et al. 2020; Denyer and Timmermans 2022). The number of expressed genes per cell at a particular time can vary. For instance, in well-characterized and differentiated human cells, it can range from a few thousand to the entire transcriptome (20,000 to 24,000 genes) (Ramsköld et al. 2009). So, other factors must also be considered, such as developmental stage, environmental conditions, and cell type etc.

## Bioinformatics tools and pipelines

The standard pipeline for single-cell long-read analyses involves quality control, read mapping, alignment correction, Cell barcode and UMI processing, gene expression quantification, batch effect correction, normalization, imputation, dimensionality reduction, feature selection, cell type clustering and annotations. The raw data for ONT long-reads are mostly stored as *.fast5* files and are 5–7 fold larger in size than raw data for short-reads with same depth which are converted and stored as *.fastq* files. For ONT data particularly, there needs to be a “base calling” step that would convert these *.fast5* files into *.fastq* files. However, there have been attempts made to reduce the size of the ONT produced raw data (Gamaarachchi et al. 2022). Furthermore, there are tools that can utilise the native (raw) ONT data as input, currently tested for smaller genomes. Here we will discuss some similarities and differences between long and short read scRNA-Seq data analysis as well as popular specialised tools designed to work with long-read scRNA-Seq. Therefore, while data processing downstream is not vastly different between long-read and short read scRNA-Seq alignment and count matrix generation require specialised tools for long-read analysis.

### Data processing of short-read vs long reads RNA-Seq

Alignment of long reads is more challenging due to the inherent higher error rates in long-read technologies, especially for ONT (Križanović et al. 2018). Specialized aligners like minimap2 (Li 2021) are often used, which can handle insertions, deletions, and other errors present in long reads, while for short reads, splice-aware aligners such as HISAT2 (Kim et al. 2019) or STAR (Dobin et al. 2013) can efficiently be used. Transcriptome assembly using long reads enables the identification of novel transcript & alternative splicing events (Moreno-Santillán et al. 2019), identification of foreign RNAs, intra-species gene-fusion transcripts. Some pipelines like SQANTI3 and FLAIR use combination of alignment-based (STAR/Kallisto and Minimap2 respectively) and subsequent de novo assembly to collapse long reads and get isoforms (Tang et al. 2020; Pardo-Palacios 2024). Conversely, other pipelines, such as RNA-Bloom2, employ a reference-free transcriptome assembly approach (Nip et al. 2023). Differential expression (DE) analysis is a common downstream step to identify genes with statistically significant changes in expression between conditions.

### Data processing of long-read for bulk RNA-Seq vs scRNA-Seq

Compared with bulk RNA-Seq, scRNA-Seq requires additional steps like cell barcode processing to identify transcripts originating from individual cells and filtering out empty droplets (containing no cells) or cells with low RNA content. Due to poor per-base sequencing accuracy in long reads, long-reads based tools strategies differ from short reads (explained below). Additionally, some protocols that involve Unique Molecular Identifier (UMI) require processing of it to account for amplification bias. Although, the same aligner can be used for both bulk and single cell, however the presence of cell barcodes and UMIs within the reads require the aligner to be able to handle them effectively (Kaminow et al. 2021). Some short-read aligners specifically designed for scRNA-Seq (e.g., STARsolo (Kaminow et al. 2021), CeleScope (https://github.com/singleron-RD/CeleScope), kallisto | bustools (Melsted et al. 2021)) incorporate functionalities to account for these sequences (with UMI and Barcodes) during the alignment process (Table 3). Processing barcode from long-read sequencing data is bit challenging especially from ONT, however this is improved significantly with time. We discussed long read bar-code processing in details below.

**Table 3:** Different aspects of short-read and long read Seq

Aspect	Short-read data analysis	Long-read data analysis
Read length	Typically < 300 base pairs	Typically > 10,000 base pairs
Sequencing technology	Illumina, Ion Torrent, etc	PacBio, Oxford Nanopore, etc
Error rate	Lower	Higher compared to short reads
Coverage	Higher	Lower
Applications	RNA-Seq, SNP calling, small indel detection	Structural variant detection, genome assembly, phasing
Complexity of analysis	Often simpler due to shorter reads	Generally more complex due to longer reads and higher error rate
Computational resources	Less computational resources required	More computational resources required
Cost	Lower cost per base	Higher cost per base
Hybrid Approaches (de-novo and reference based)	Commonly used in hybrid assembly methods	Less common due to inherent advantages of long reads

Downstream analyses specific to single-cell RNA sequencing (scRNA-seq) delve deeper into cellular heterogeneity. Techniques such as dimensionality reduction (e.g., PCA, UMAP, tSNE) and cell-type clustering enable researchers to pinpoint distinct cell populations within the sample. Cell-type annotation is typically achieved through reference-based methods [e.g., singleR (Aran et al. 2019), scPred (Alquicira-Hernandez et al. 2019), scClassify (Lin et al. 2020)], and leveraging known cell type markers identified by tools like Seurat and sctype. Additionally, popular analyses include differential cellular composition (DCC) and cell–cell interaction (CCI) studies. DCC analyses identify cell types with statistically significant changes in abundance across multiple experimental conditions. CCI provides insights into active regulatory networks within cell subpopulations, shedding light on the mechanisms driving cellular heterogeneity.

### Cell barcode/UMI assignment for long-read scRNA-Seq

Recovering accurate cell barcodes and UMIs from single-cell ONT sequencing data poses a significant challenge when dealing with poor per-base sequencing accuracy, typically ranging from 87 to 95% (Philpott et al. 2021). This issue arises because short sequence tags, such as barcodes, are highly sensitive to sequencing errors (Philpott et al. 2021). Currently used strategies include (a) Iterative sequencing, for error reduction (Sameith et al. 2017), (b) Modifying the read structure to increase error tolerance (Philpott et al. 2021), and (c) Employing hybrid sequencing (short-read and long-read) techniques (Sović et al. 2016), where short reads used to create reference barcode sets for the extraction of cell barcodes and UMIs. For the creation of reference cell barcode list, a shallow short-read sequencing run is acceptable; however, highly saturated sequencing can be expensive, is necessary for the creation of a reference UMI list. Following are some computational methods developed to provide solution for this challenge.

SiCeLoRe (Single Cell long-read) is a set of tools for the highly multiplexed single-cell Nanopore or PacBIo long-read sequencing data that are used for cell barcode/UMI assignment and bioinformatics analysis (Lebrigand et al. 2020). The workflow incorporates several sequential steps for cell barcode and UMI assignment to long-read (guided by short-read data), transcript isoform identification, generation of molecules consensus sequences (UMI-guided error-correction), and production of [isoforms/junctions/SNPs x cells] count matrices for new modalities integration into standard single-cell RNA-Seq statistic.

Currently, BLAZE (You et al. 2023) can only recognize 10 × single-cell barcodes from nanopore scans. Although various LR single-cell techniques have been utilized to profile single cells with long-read, including scCOLOR-seq and R2C2, the 10 × chromium platform is the most accessible and well-liked. For Nanopore long-read, BLAZE displays an accurate single-cell barcode recognition tool. BLAZE can integrate downstream gene/isoform identification and quantification and that it performs well across a variety of data sets, read depths, and read accuracies. Importantly, BLAZE overcomes the requirement for extra matched short-read (SR) data, simplifying LR scRNA-Seq methods while drastically lowering cost.

ScNapBar (single-cell Nanopore barcode demultiplexer) demultiplexes Nanopore barcodes and is particularly suited for low-depth Illumina and Nanopore sequencing (Wang et al. 2021b). The high error rate of Nanopore reads poses a challenge for cell barcode assignment. This method propose a solution to this problem by using a hybrid sequencing approach on Nanopore and Illumina platforms.

### Isoform and gene fusion analyses at single cell level

Several specialized tools have been developed to handle single-cell long-read data for isoform identification and downstream analysis. Examples include SiCeLoRe (Philpott et al. 2021), FLAMES (Holmqvist et al. 2021), and FLAIR (Tang et al. 2020). These tools are specifically designed to work with long-read data and facilitate isoform-level analysis. Additionally, tools like JAFFAL (Davidson et al. 2022) are dedicated to identifying gene fusions in single-cell long-read data. Other tools like scTagger and FLAIR are specialized in detecting RNA splicing events from single-cell long-read data.

### Inferencing cell trajectories at isoform level

Data obtained using Long-read sequencing at single cell level provides a good representation of isoform diversity which can help to better infer cell trajectories at isoform level. Since many RNA velocity models relies on the assumption of constant splicing rate of pre-mRNAs over time, which may not be true for complex splicing patterns (Wu and Schmitz 2023). In context to long-read scRNA-Seq, there is a scope to develop tools that integrate the complex splicing mechanisms to construct comprehensive mathematical models of cell-fate determination. However, long-read based tools like FLAMES provide deeper insights into isoform expression dynamics and splicing at the single-cell level. Moreover, FLAMES offers the capability to detect mutations in single-cell long-read data. This tool, along with others as mentioned in Table 4, contributes to the comprehensive analysis of single-cell long-read data, enabling various functional insights.

**Table 4:** Bioinformatics tools developed for the analysis of long-read scRNA-Seq

Tool	Sc RNA	Long reads	Category	Feature/s	References
JAFFAL	✔	✔	Gene fusion detection	Gene fusion detection	Davidson et al. (2022)
scTagger	✔	✔	RNA splicing, barcodes	Better accuracy and time efficiency	Ebrahimi et al. (2022)
R2C2	✔	✔	Full length isoform identification	Generate consensus sequences with increased base accuracy	Volden et al. (2018)
FLAMES	✔	✔	Full length Iso-form Identification, quantification of full-length splice variants	Isoform discovery, splicing analysis, and mutation detection	Holmqvist et al. (2021)
SiCeLoRe	✔	✔	Full length Isoform Identification, Barcodes	Cell barcode/UMI (unique molecular identifier) assignment	Lebrigand et al. (2020)
ScISOr-Seq	✔	✔	Full-length Isoform Identification	Identifies single-cell types and their full-length RNA isoforms	Gupta et al. (2018)
Longcell	✔	✔	Isoform quantification	Rigorously quantifies the level of inter-cell versus intra-cell diversity in exon usage	Fu, et al. (2023)
Mandalorion	✔	✔	Full length Isoform Identification and quantification	LRGASP consortium challenge shows it has high Precision and Recall	Volden et al. (2022)
BLAZE	✔	✔	Barcodes	10 × cell barcodes using only nanopore LR scRNA-Seq data. No paralleled short-read data needed, but theoretical barcode whitelist required	You et al. (2023)
ScNapBar	✔	✔	Barcodes	Enables cell barcode assignment with high accuracy, especially if sequencing saturation is low	Wang et al. (2021b)
scCOLOR-seq	✔	✔	Barcodes	Enables error correction of barcode and UMI oligonucleotide sequences	Philpott et al. (2021)
wf-single-cell (Nanopore)	✔	✔	Barcodes	Compatible with nanopore sequencing reads generated from single-cell gene expression libraries	https://github.com/epi2me-labs/wf-single-cell
scNanoGPS	✔	✔	Identifies cell-type-specific isoforms and mutations in addition to gene expression profiles, enabling synchronous cell-lineage (genotype) and cell-fate (phenotype) tracing	Without short read nor whitelist guidance	Shiau et al. (2023)

## Prospects of long-read single cell sequencing

Long-read scRNA-Seq is undoubtedly poised to revolutionize the field of transcriptomics by providing data that can be used for a wide range of computational studies, including isoform and gene fusion analyses at the single-cell level. The exploration of isoform and alternative splicing events at the single-cell level is particularly intriguing, as this high-resolution data enables deep investigation of cell-to-cell variations and useful to study the role of alternative splicing in heterogenous population formation. Additionally, long-read scRNA-Seq has the potential to detect RNA modification at single cell level.

The development of novel long-read single-cell library preparation methods, along with precise bioinformatics tools and robust mathematical models, is imperative for the effective production, processing, and analysis of long-read scRNA-Seq data. These advances will enhance our ability to dissect complex biological mechanisms, including those underlying development and disease progression. This progress promises a comprehensive exploration of isoform diversity and alternative splicing, significantly expanding our understanding of cellular processes.

## Data Availability

There is no data associated with this manuscript.

## Abbreviations

ONT: Oxford Nanopore Technology
UMI: Unique molecular identifiers
TSO: Template switching oligo
